# Supplementary material for: Long-term implicit memory for sequential auditory patterns in humans
Source: eLife. 2020 May 18;9:e56073. doi: 10.7554/eLife.56073 (PMC7338054; doi:10.7554/eLife.56073)
Supplement: Transparent reporting form [file elife-56073-transrepform.pdf]

eLIFE
1st Floor
24 Hills Road
Cambridge CB2 1JP, UK
P 01223 855340
W elifesciences.org
T @elife

eLife's transparent reporting form

We encourage authors to provide detailed information *within their submission* to facilitate the interpretation and replication of experiments. Authors can upload supporting documentation to indicate the use of appropriate reporting guidelines for health-related research (see EQUATOR Network), life science research (see the BioSharing Information Resource), or the ARRIVE guidelines for reporting work involving animal research. Where applicable, authors should refer to any relevant reporting standards documents in this form.

If you have any questions, please consult our Journal Policies and/or contact us: editorial@elifesciences.org.

Sample-size estimation

- You should state whether an appropriate sample size was computed when the study was being designed
- You should state the statistical method of sample size computation and any required assumptions
- If no explicit power analysis was used, you should describe how you decided what sample (replicate) size (number) to use

Please outline where this information can be found within the submission (e.g., sections or figure legends), or explain why this information doesn't apply to your submission:

The sample size was estimated with a power calculation based on data acquired in a pilot experiment. Detailed information is reported in the Methods section under the 'Participant numbers' paragraph. Details about sample size for each experiment can be found in the Methods section under each experiment description.

Replicates

- You should report how often each experiment was performed
- You should include a definition of biological versus technical replication
- The data obtained should be provided and sufficient information should be provided to indicate the number of independent biological and/or technical replicates
- If you encountered any outliers, you should describe how these were handled
- Criteria for exclusion/inclusion of data should be clearly stated
- High-throughput sequence data should be uploaded before submission, with a private link for reviewers provided (these are available from both GEO and ArrayExpress)

Please outline where this information can be found within the submission (e.g., sections or figure legends), or explain why this information doesn't apply to your submission:

eLife Sciences Publications, Ltd is a limited liability non-profit non-stock corporation incorporated in the State of Delaware, USA, with company number 5030732, and is registered in the UK with company number FC030576 and branch number BR015634 at the address 1st Floor, 24 Hills Road, Cambridge CB2 1JP | August 2014

We report 9 different experiments. Each includes a replication of the main finding as well as additional manipulations. We also include a meta-analysis (pooling data across all studies) which estimates the effect sizes and replicability across subjects (both, very high).

Individual data are plotted in each figure (+ the data are uploaded with the submission).

The details of the statistical tests are explained in the method section.

Sample sizes are defined in the figures relative to each experiment and in the Methods section under each experiment description.

Information on outliers, and exclusion/inclusion criteria can be found in Methods ('Statistical analysis' paragraph). Participants couldn't take part in more than one experiment. Participants were excluded based on their general sensitivity to the presence of regularities (see Methods for details).

Statistical reporting

- Statistical analysis methods should be described and justified
- Raw data should be presented in figures whenever informative to do so (typically when N per group is less than 10)
- For each experiment, you should identify the statistical tests used, exact values of N, definitions of center, methods of multiple test correction, and dispersion and precision measures (e.g., mean, median, SD, SEM, confidence intervals; and, for the major substantive results, a measure of effect size (e.g., Pearson's r, Cohen's d)
- Report exact p-values wherever possible alongside the summary statistics and 95% confidence intervals. These should be reported for all key questions and not only when the p-value is less than 0.05.

Please outline where this information can be found within the submission (e.g., sections or figure legends), or explain why this information doesn't apply to your submission:

Statistical methods relevant for each experiment are described in the Methods section ('Statistical analysis').
Single participants data are presented in the figures pertaining to each experiment.
As explained in the Methods ('Statistical analysis'), for the omnibus tests p-values were Greenhouse-Geisser adjusted when sphericity assumptions were violated and partial eta square values are reported as a measure of the effect sizes. The Bonferroni method for multiple test correction was applied when necessary.

(For large datasets, or papers with a very large number of statistical tests, you may upload a single table file with tests, Ns, etc., with reference to sections in the manuscript.)

Group allocation

- Indicate how samples were allocated into experimental groups (in the case of clinical studies, please specify allocation to treatment method); if randomization was used, please also state if restricted randomization was applied
- Indicate if masking was used during group allocation, data collection and/or data analysis

Please outline where this information can be found within the submission (e.g., sections or figure legends), or explain why this information doesn't apply to your submission:

N/A (All experiments are based on a repeated-measures design)

Additional data files ("source data")

- We encourage you to upload relevant additional data files, such as numerical data that are represented as a graph in a figure, or as a summary table
- Where provided, these should be in the most useful format, and they can be uploaded as "Source data" files linked to a main figure or table
- Include model definition files including the full list of parameters used
- Include code used for data analysis (e.g., R, MatLab)
- Avoid stating that data files are "available upon request"

Please indicate the figures or tables for which source data files have been provided:

All behavioral datasets and modelling script, for this study, along with relevant documentation, can be found in the OSF repository (https://osf.io/dtzs3/; DOI 10.17605/OSF.IO/DTZS3).<br><br>(this statement is also included in the manuscript)
